# A review on the clean-up technologies for heavy metal ions contaminated soil samples

**DOI:** 10.1016/j.heliyon.2023.e15472

**Published:** 2023-04-15

**Authors:** Vikas Kumar, Chadetrik Rout, Joginder Singh, Yajvinder Saharan, Rohit Goyat, Ahmad Umar, Sheikh Akbar, S. Baskoutas

**Affiliations:** a Department of Civil Engineering, Maharishi Markandeshwar (Deemed to Be University), Mullana, Ambala, 133203, Haryana, India; b Department of Chemistry, Maharishi Markandeshwar (Deemed to Be University), Mullana, Ambala, 133203, Haryana, India; c Department of Chemistry, Faculty of Science and Arts, And Promising Centre for Sensors and Electronic Devices (PCSED), Najran University, Najran-11001, Kingdom of Saudi Arabia; d Department of Materials Science and Engineering, The Ohio State University, Columbus, OH 43210, USA; e Department of Materials Science, University of Patras, Patras, Greece

**Keywords:** Heavy metal ions, Lung cancer, Remediation technology, Contaminated soil, Permissible limits, And feasibility

## Abstract

The soil contamination with heavy metal ions is one of the grave intricacies faced worldwide over the last few decades by the virtue of rapid industrialization, human negligence and greed. Heavy metal ions are quite toxic even at low concentration a swell as non-biodegradable in nature. Their bioaccumulation in the human body leads to several chronic and persistent diseases such as lung cancer, nervous system break down, respiratory problems and renal damage etc. In addition to this, the increased concentration of these metal ions in soil, beyond the permissible limits, makes the soil unfit for further agricultural use. Hence it is our necessity, to monitor the concentration of these metal ions in the soil and water bodies and adopt some better technologies to eradicate them fully. From the literature survey, it was observed that three main types of techniques viz. physical, chemical, and biological were employed to harness the heavy metal ions from metal-polluted soil samples. The main goal of these techniques was the complete removal of the metal ions or the transformation of them into less hazardous and toxic forms. Further the selection of the remediation technology depends upon different factors such as process feasibility/mechanism of the process applied, nature and type of contaminants, type and content of the soil, etc. In this review article, we have studied in detail all the three technologies viz. physical, chemical and biological with their sub-parts, mechanism, pictures, advantages and disadvantages.

## Graphical abstract

**Figure:**
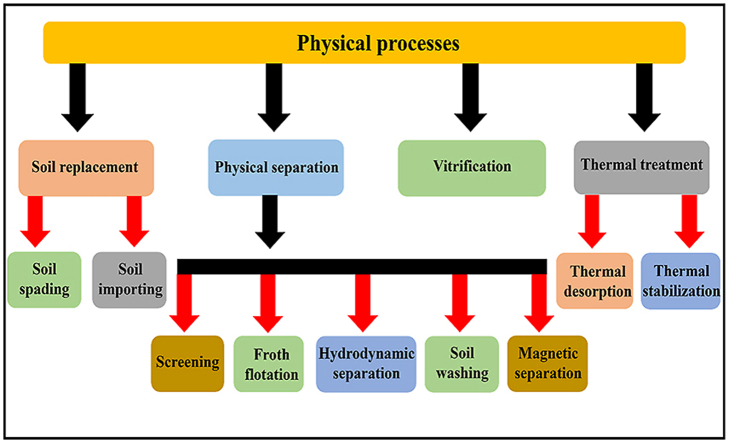

## 1 Introduction

The air, water, and soil are the most important components of the environment on this planet, which need to be protected and preserved so that the present and coming generations of the biotic community can survive and prosperous [[1], [2], [3]]. However, the rapid increase in population worldwide resulted in speedy industrialization which in one or another way have led to stern contamination of our air, water, soil and other natural resources [[4], [5], [6]]. In addition to this, a lack of proper knowledge of effluent dumping and failure to imply firm regulatory standards have added to the cause of environmental worsening and deterioration [7,8].

**Fig. 1:**
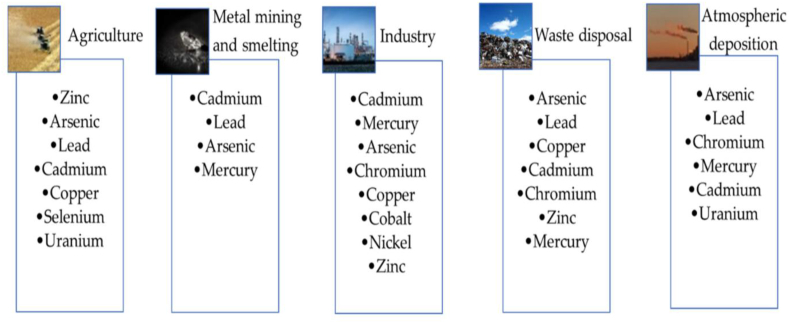
The list of major heavy metals thrown from different sectors leading to soil pollution. [Reprinted with permission from the reference number [11] T. K. Das, A. Poater, Review on the Use of Heavy Metal Deposits from Water Treatment Waste towards Catalytic Chemical Syntheses. *Int. J. Mol. Sci.*, 2021, **22**, 13383, Copyright @ MDPI].

Further the excessive use of fertilizers, pesticides and herbicides to increase the crop production in the last few decades is considered an ill practice by the farmers which have desirably or undesirably polluted the soil with numerous heavy metals viz. Cd, Cu, Ni, Zn, Hg, As and Pb etc [9,10]. The majorheavy metals which contaminate the soil come from agriculture products, waste disposal, mining and industrial sectors are detailed in Fig. 1.

**Fig. 2:**
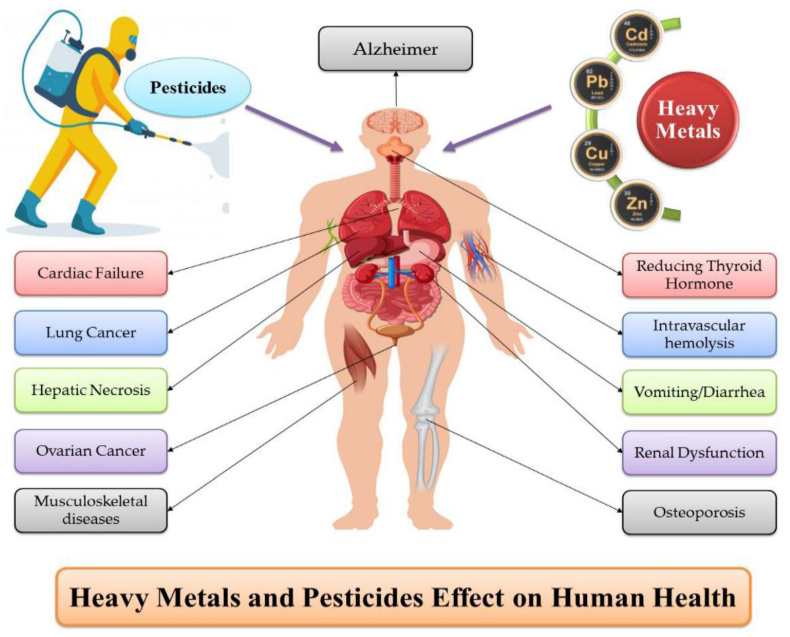
The effect of HMIs and pesticides on human health [Reprintedwith permission from the reference number [8] Alengebawy, A., Abdelkhalek,S. T., Qureshi, S. R., & Wang, M. Q., Heavy metals and pesticide toxicity inagricultural soil and plants: Ecological risks and human health implications, *Toxics*, 2021, **9**(3), 42. Copyright @ MDPI].

In long term, these metal ions have entered the food chain leading to several diseases such as lung cancer, nervous system break down, vomiting, muscle cramps, respiratory problems and renal damage etc [8]. The details of the diseases along with pesticides and metal ions are given in Fig. 2.

In addition to this Table 1, further highlights the properties, applications and permissible limitin mg/Lof different metal ions as guided by WHO/UPEA [12].

**Table 1:** Physical, chemical properties, applications and permissible limit (WHO/UPEA) mg/Lof heavy metal ions.

Heavy metals	Physical and chemical properties	Applications of heavy metals	Permissible limit (WHO/UPEA) mg/L in water and soil
Copper	Atomic mass = 63.55	Electronic and electrical, transport equipment, industrial machinery, construction, pesticides	2.0/1.3
	Density = 8960 kg/m^3^		
	Heat of fusion = 13.26 kJ/mol		
	Colour = Reddish-brown		
Zinc	Atomic mass = 65.38s	Rubber, cosmetics, paints, plastic, soaps, inks, pharmaceuticals, textiles, batteries and electrical equipment	3.0/5.0
	Density = 7140 kg/m^3^		
	Heat of fusion = 7.32 kJ/mol		
	Colour = Bluish-white		
Cadmium	Atomic mass = 112.41	Silver-cadmium batteries, plastics, electroplating, coating operations, paints pigments, television phosphors, photography, machinery and baking enamels	0.003/0.005
	Density = 8650 kg/m^3^		
	Heat of fusion = 6.21 kJ/mol		
	Colour = Silver-white		
Chromium	Atomic mass = 51.99	Paint pigments, industrial applications, catalysts, tanning agents, photography and alloys	0.05/0.1
	Density = 7190 kg/m^3^		
	Heat of fusion = 21.00 kJ/mol		
	Colour = Silver-gray		
Lead	Atomic mass = 207.2 Density = 11,340 kg/m^3^	Building construction, cable coatings ammunition, electrical accumulators and batteries	0.01/0.005
	Heat of fusion = 4.77 kJ/mol		
	Colour = Silver-white		
Arsenic	Atomic mass = 74.92	Semiconductors, alloys, pharmaceuticals and pesticides	0.01/0.05
	Density = 5730 kg/m^3^		
	Heat of fusion = 24.44 kJ/mol		
	Colour = Silver-gray		
Mercury	Atomic mass = 200.59	Thermometers, pharmaceuticals, dental preparation, fungicides, seed dressings, fluorescent and ultraviolet lamps	0.001/0.002
	Density = 13,530 kg/m^3^		
	Heat of fusion = 2.29 kJ/mol		
	Colour = Slate-gray		
Nickel	Atomic mass = 56.69	Used in making coins, gas turbines, rocket engines and alloys	0.02/0.05
	Density = 8908 kg/m^3^		
	Heat of fusion = 17.2 kJ/mol		
	Colour = Silver-white		

It was observed from Table 1 that metals such as Cadmium, Chromium, Zinc, Nickel, Arsenic, Mercury, lead and copper have permissible limits of less than 0.01 ppm/L, which is quite low. It is a matter of great concern and needs to be addressed as soon as possible.

This review article highlights in detail all the three technologiesviz. physical, chemical and biological alongwiththeir sub-parts, mechanism, advantages and disadvantages.

## 2 Different techniques employed for the removal of HMIs fromHMIscontaminated soil samples

Over the last few decades, the three main types of techniques viz. physical, chemical, and biological were employed to harness the heavy metal ions from metalpolluted soilsamples [13,14]. The main goal of these techniques was the complete removal of the metal ions or the transformation of them into less hazardousand toxic forms. These techniques differ in terms of the mechanism applied to remove/degrade the pollutants from soil [15]. Further, these techniques can be also characterized by the location where they are employed, viz. in-situ, ex-situ and on site. In the in-situ method, the treatment technology is directly performed on the location where there is metal ions pollution, without excavating or moving the soil from the site. On the other hand, in on-site methodology, the soil is taken away from the site and treatedinsome other surrounding or nearby place. The technique can be carefully monitored and kept under control. Inex-situ: the remediation occurs at a site far from the polluted area, and this entails soil excavation, its transport to a processing plant, and often transport back to the original site [16,17].

For some techniques, the operation can be carried out at all kinds of locations, whereasfor others it can occur only in-situ. Each location involves different features and impactsthat are typical of the considered polluted site [18]. Therefore, a detailed discussion with anassessment of the pros and cons is must.The Table 2 highlights the sub-parts of the three main technologies with their maximum removal efficiency, advantages and disadvantages.

**Table 2:** Different remediationprocesses for heavy metals removal.

Sr. No.	Remediation technology		Maximum removal efficiency (%)	Advantages	Disadvantages	Location	Reference
1.	Physical	Physical separation	95	Fast and simple process with high removal efficiency	Required high cost	X, Y, Z	[[16], [17], [18], [19]]
		Thermal treatment				X, Y, Z	[[20], [21]]
		Vitrification				X	[22]
2.	Chemical	Stabilization	90	Fast and simple process with high removal efficiency	Change the physiochemical properties of soil and required high cost	X, Y, Z	[[23], [24]]
		Treatment using nano-technology				Y, Z	[[25], [26], [27], [28], [29], [30], [31], [32], [33], [34]]
		Stabilization/solidification				X	[35]
		Soil washing using specific chemical				Y, Z	[[36], [37], [38]]
		Electrochemical remediation				X	[[39], [40], [41], [42], [43]]
3.	Biological	Biosorption	96	Simple, cost-effective, and eco-friendly	Slow remediation process and only applicable for low contaminated sites	X, Y, Z	[[44], [45], [46], [47], [48], [49], [50], [51], [52]]
		Bioleaching				Y, Z	[[53], [54]]
		Phytoremediation				X	[[55], [56], [57], [58], [59], [60], [61], [62], [63], [64], [65], [66], [67], [68], [69], [70]]

X: in situ treatment, Y: on site treatment and Z: ex situ treatment.

### 2.1 Physical processes

**Fig. 3:**
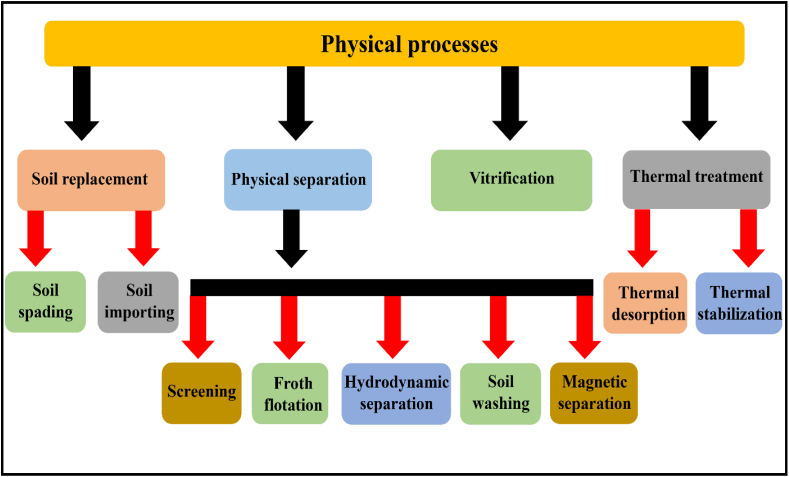
Physical remediation processes to remove HMIsfrom metal-contaminated soil samples.

In this remediation process, the metal-polluted soil isconverted into less toxic via various physical mechanismssuch asphysical separation, soil replacement, vitrification, and thermal treatments as shown in Fig. 3. These remediation methods have some unique characteristics such as they are quite simple, fast, and have high metal ion removal capacity [[16], [17], [18], [19], [20]].

#### 2.1.1 Physical separation

The physical separation technique is employed in the mining and mineral processing industry for the uptake of metal ions from metal-polluted sites at a higher scale [17]. Basically, this remediation process depends uponthe nature of the soil such as clay content, humic content, particle size, shape, moisture content, as well as metal ions density difference [71]. Further, this technique is subdivided intofive different types viz.; screening, froth flotation, hydrodynamic separation, soil washing and magnetic separation.

##### 2.1.1.1 Screening

In this methodology, the soil particles are vibrated and sieved through the screens having different mess size [71]. Thesoil particle sizeas well as the vibration rate affects the overall metal ion separation. In the year 2015, Umar and Halimoonused this screening method for the uptake of copper, chromium, lead and cadmium metals from metal ions contaminated sites with 50 mg/L metal ions removal efficiency [72]. SimilarlyWolowicz and Wawrzkiewiczin 2021 applied the screening process for the removal of Ni (II) metal ions from the aqueous solution at different concentrations of HCl andHCl/HNO_3_ [73].

##### 2.1.1.2 Hydrodynamic separation

The hydrodynamic separation depends upon the velocity with which particles fall through the water flow or the applied various centrifugal force to the water flow. The centrifugal force is more powerful than the gravity force and easilyseparates fine soil particles from large sand particles [71]. Further, this process also reduced the separation operating time. Khulbe and Matsuura 2017 used the hydrodynamic separation method for the removal of heavy metal ions from the metal-polluted sites [74]. Fato et al., (2019) applied this process for the uptaking of metal ions from river water and uptake up to 98% indivisibly metal ions [75].

##### 2.1.1.3 Froth flotation

**Fig. 4:**
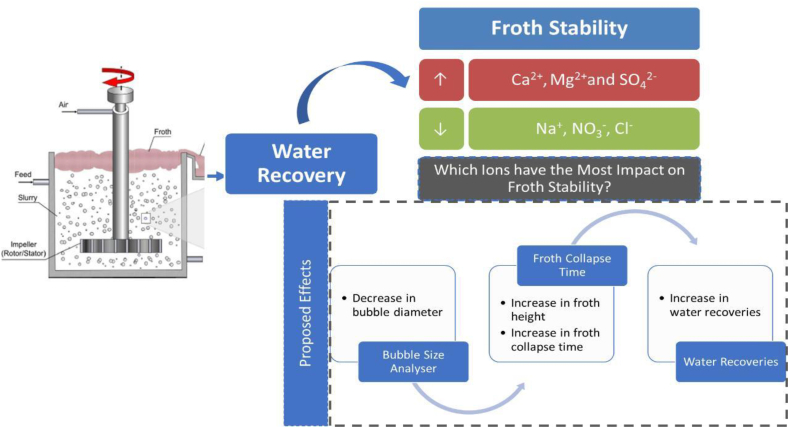
Schematic diagrams of the froth flotation process [Reprinted with permission from the reference number [78] Manono MS, Corin KC. Considering Specific Ion Effects on Froth Stability in Sulfidic Cu–Ni-PGM Ore Flotation. Minerals. 2022 Mar 4; 12(3):321, Copyright @ MDPI].

In this technique, the air is injected into the slurry of metal polluted soil resulting in the formation of froth. The froth formed rises to the surface of the slurry and removed the metal contaminants as shown in Fig. 4 [71]. The separation process depends upon the binding affinity of a particle hydrophobic surface for an air bubble injected in the slurry of soil. Further, the addition of surfactants increases the hydrophobic nature of metal-bearing particles and enhances the removal efficiency. Cauwenberg et al., (1998) investigated that the heavy metal was removed up to 80% at different pH in the froth flotation process [76]. Bergeron et al., (2001) applied the froth flotation process for the separation of the Cd, Cu, Pb, and Zn metals from contaminated soil samples [77].

##### 2.1.1.4 Magnetic separation

**Fig. 5:**
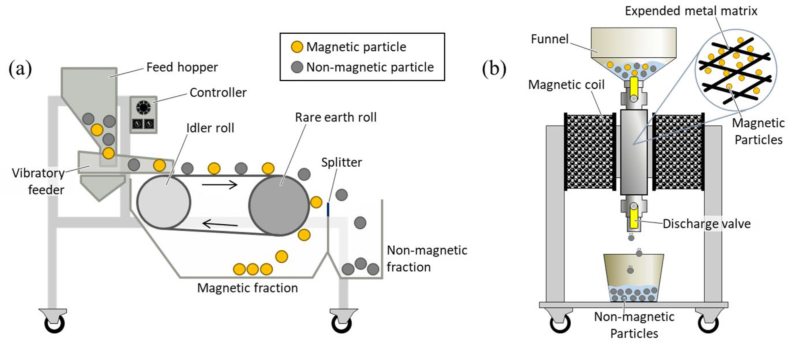
Schematic diagrams of (**a**) dry high-intensity magnetic separator and (**b**) wet high-intensity magnetic separator [Reprinted with permission from the reference number [82] Park, I.; Kanazawa, Y.; Sato, N.; Galtchandmani, P.; Jha, M.K.; Tabelin, C·B.; Jeon, S.; Ito, M.; Hiroyoshi, N. Beneficiation of Low-Grade Rare Earth Ore from KhalzanBuregtei Deposit (Mongolia) by Magnetic Separation. Minerals 2**021**, 11, 1432, Copyright @ MDPI].

Magnetic separation is a process, in which magnetic force is applied to extract the metal from the contaminated soil as shown in Fig. 5. The metal removal efficiency is based on their diverse magnetic characteristics. Because, the particles present in the soil have different magnetic susceptibility range from negative (organic), and intermediate (paramagnetic) to positive ferromagnetic minerals [71]. The ferromagnetic material can be separated using a low magnetic field, while the paramagnetic material separates by a high magnetic field. Knott et al., (2007) used low-intensity magnetism for the recovery of metal debris from military sites [79]. In addition, Feng et al., (2007) also found that more than 95% of Cu metal was removed from contaminated soil in magnetic separation after applying 5% iron filling for 3 h [80]. Jeong et al., (2021) used the magnetic separation method for cleanup of metals viz. Cu, Zn, Cd, Ni, Cr, and Mn from road dust industrial areas [81]. It not only reduced the metal contaminants from the industrial area but also reduces waste generation.

##### 2.1.1.5 Soil washing

**Fig. 6:**
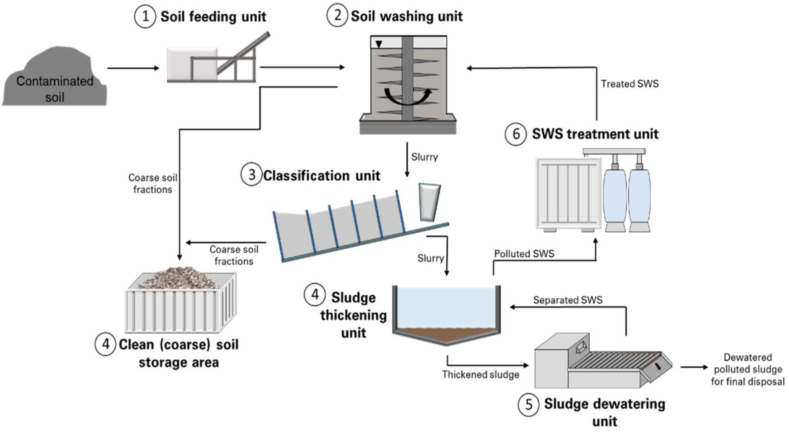
Soil washing process for metal/metalloids contaminated soil [Reprintedwith permission from the reference number [83] Dekonta Transportable Soil Washing Plant. Available online: https://www.dekonta.cz/en/about-us/download.html(accessed on May 29, 2020) Copyright @ MDPI].

The soil washing processis anex-situ process, in which physical and chemical procedures are applied to the extraction of HMIs from polluted soil samples as shown in Fig. 6. In the physical process, the metal atoms removal efficiency depends upon the physical characteristic of metal atoms as well as soil particles [71]. While in the case of a chemical process the solubility of the metal atom depends upon the fluid containing acids and chelating agents. Yi et al., (2015) employed different physical separation processes and extraction reagents for the removal of Pb and Zn metal from the polluted sites [19]. Further, Liao et al., (2016) obtained a high removal efficiency of heavy metal in the small particle size fraction [17]. Boente et al., (2017) used the soil washing technique to remediate the heavy metal (As, Cu, Hg, Pb, and Sb) from a brown field affected by pyrite ash disposal [16]. In addition to this, Park et al., (2017) investigated the combined effect of the ultrasonic and mechanical soil-washing process and found that ultrasound enhances the heavy metal removal efficiency in less favourable conditions for the mechanical processes [18].

##### 2.1 2 Soil replacement

**Fig. 7:**
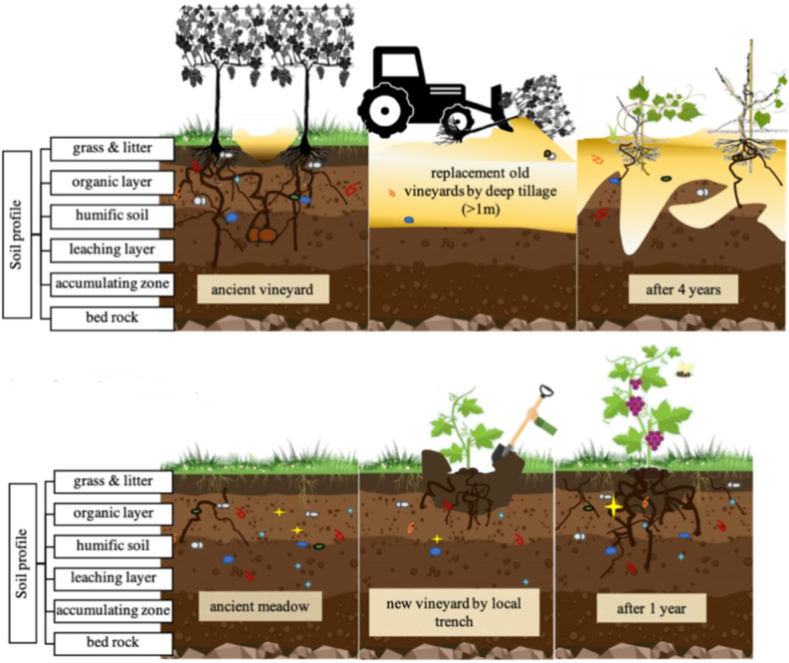
(A)Cross-sectional image showing effect of the soil replacement [Reprinted with permission from the reference number [89] Gagnarli, E.; Valboa, G.; Vignozzi, N.; Goggioli, D.; Guidi, S.; Tarchi, F.; Corino, L.; Simoni, S. Effects of Land-Use Change on Soil Functionality and Biodiversity: Toward Sustainable Planning of New Vineyards. Land **2021**, 10, 358, Copyright @ MDPI].

In this technique, either the partial or the total replacement of polluted soil with unpolluted soil decreases the HMIs/metalloid concentration in contaminated sites as shown in Fig. 7. Further, the replaced soil is treated as waste [84]. This method is only applicable to small-scale treatment. The soil replacement process is further of two types such as soil spading and soil importing. In the soil spading mechanism, the polluted soil is dug entirely, spaded, and then replaced with new (clean) soil. But in the case of soil importing, the fresh soil is mixed with contaminated soil to decrease the HMIs concentrations. Before replacement, the polluted area should be isolated from the surrounding via physical barriers to avoiding the contaminants of the neighbouring area [85]. Douay et al., (2008) investigated the cadmium, lead, and zinc metals removal from the urban contaminated soils in north France using the soil replacement method [86]. Han et al., (2019) studied the replacement of cadmium contamination with good fertility soil, and reduce the risk of cadmium pollutionin the surrounding environment [87]. Abdullahi et al., (2021) removedthe cadmium, copper, lead, and arsenic contaminants by replacing contaminated soil with new soil [88].

#### 2.1.3 Thermal treatment

**Fig. 8:**
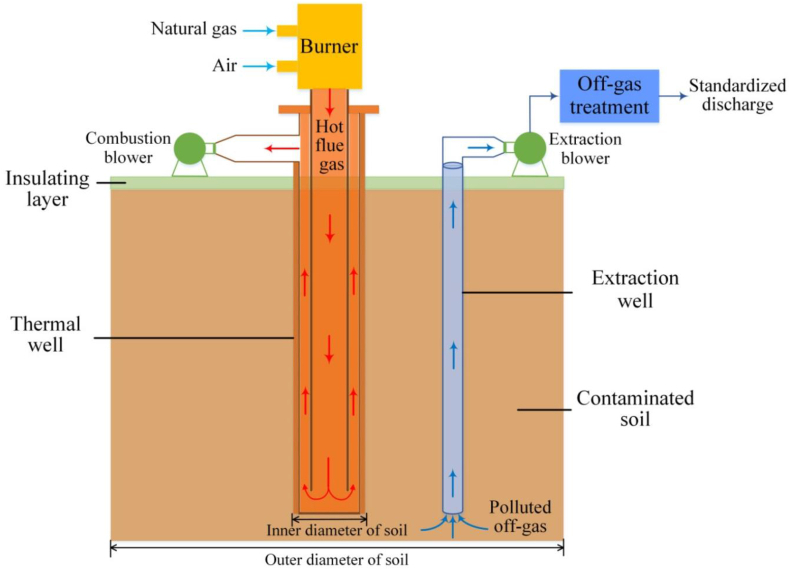
Schematic diagram of the thermal desorption system [Reprinted with permission from the reference number [95] Xu H-J, Li Y-Z, Gao L-J, Zhang X. Planned Heating Control Strategy and Thermodynamic Modeling of a Natural Gas Thermal Desorption System for Contaminated Soil. Energies. 2020; 13(3):642 Copyright @ MDPI].

In this method, heat is applied under controlled temperature conditions into metal-contaminated soil as shown in Fig. 8, which mobilizes and evaporates the volatile as well as semi-volatile substances into less hazardous substances [20]. The desorbed pollutants are collected using carrier gas or vacuum pressure. The contaminated soil can be heated by various techniques such as electric heating, radiofrequency heating, steam heating, and conducting heating. Depending upon a contaminant's boiling point, this technique can be employed at a low-temperature range (90–320 °C) as well as ata high-temperature range (320–560 °C) [21].

Chang et al., (2006) observed that when the mercury polluted soil was heated at 357 °C, the metal boils and desorbed from the soil [90]. Further, Fujun et al., (2014) studied the mercury-contaminated soil and found that 0.8 mg/kg was removed at 400 °C temperature after 60 min of treatment time [91]. Samaksaman et al., (2015) observed that 48–85% of metal was removed from the contaminated soil samples at a temperature range of 500–800 °C [92]. Wang et al., (2018) investigated that thermal stabilization reduces the mobility of Zn and Cu metal in polluted soil and found that the metal concentration was decreased when increased in temperature up to 700 °C [93]. In addition to this, Jining et al., (2018) studied the combined effect of chlorination and thermal treatment and removed the Cd and Pb metal ions from metal-contaminated soil at 950 °C temperature [94].

#### 2.1.4 Vitrification

**Fig. 9:**
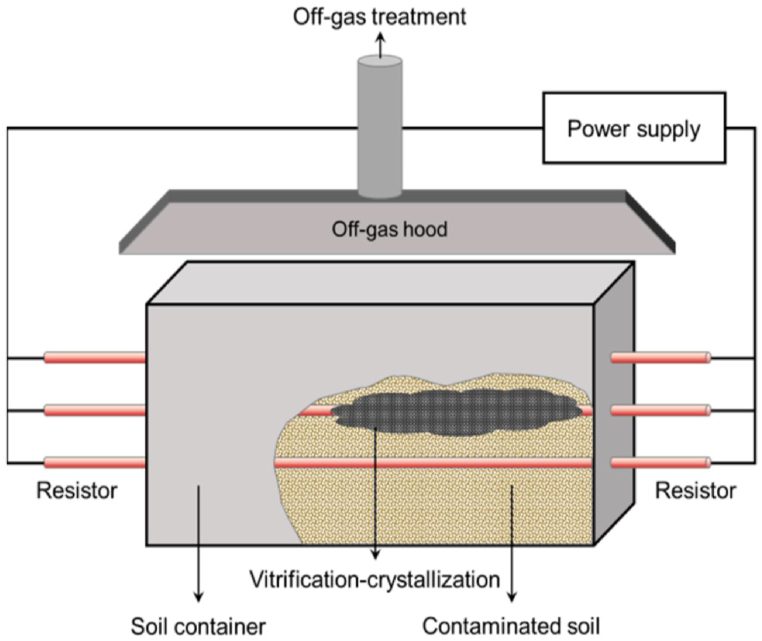
Schematic diagram of electrical resistance heating device showing vitrification [Reprinted with permission from the reference number [99] Kim, C.; Koh, T.; Lee, D.; Park, D. Assessment of Heavy Metal and Oil-Contaminated Silty Sand Treatment by Electrical Resistance Heating Method. *Appl. Sci.***2022**, *12*, 4630, Copyright @ MDPI].

This is another thermallycontrolled technique, in which the metal-contaminated soil is heated at a high temperature (>1500 °C) which decreasesthe movementof HMIs by fixing them into vitreous material. In this process, the sand is converted into a meltedlava, and on cooling it is converted into a glassy matrix, while the other pollutants are destroyed [22]. The main sources for heating the polluted soil are high voltage electricity, and electrical gas plasma. As seen in Fig. 9, by passing high electric current to flow through a resistor that was positioned in the soil, the soil volume was speedily heated, resulting in an increase in temperature, which evaporated volatile (organic) contaminants and isolated (inorganic) heavy metal contaminants as crystal structures (solidification) in soil. Navarro et al., (2013) applied the vitrification process for removal of contaminants from Pb–Ag mines in spain. It was observed thatzinc, iron, nickel, copper, and manganese are immobilization at 1350 °C [96]. Further, Dellisanti et al., (2016) investigated in-field Joule heating vitrification at 1850 °C for the removal of tons of zinc and lead metal from contaminated soil [97]. Kuo and Wu et al., (2021) studied the in-situ vitrification at 1600–2000 °C for the remediation of heavy metals from unidentified waste and groundwater treatment sites [98].

#### 2.1.5 Advantages and disadvantages of physical process

The physical process is a quite simple, fast, and effective remediation technique with high removal efficiency. The soil replacement method is suitable for small areas which are highly polluted. Further, the thermal treatment is safe, has less energy consumption, and produces little secondary pollution as compared to other methods.

The major drawbacks of this technique are that it changes soil properties viz. particle size, and soil texturewhich affects soil fertility. This remediation process requires large spaces and large equipment forsoil treatments. Further, thermal treatment requireshighcosts and gas emission control. Moreover, the vitrification process isnot applicable for soil containing high moisture and organic matter.

### 2.2 Chemical processes

**Fig. 10:**
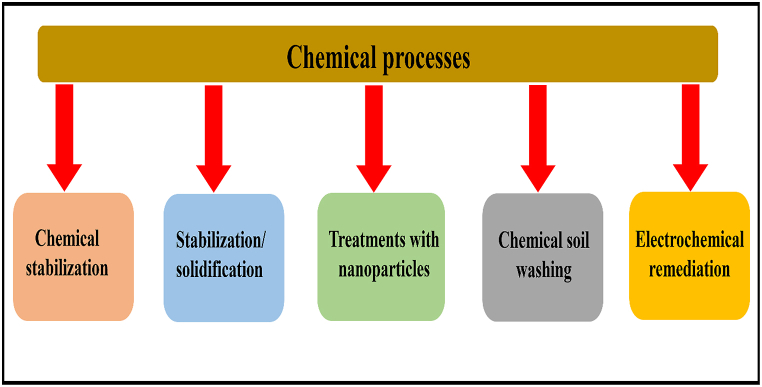
Types of chemical processes used for metal-contaminated soil.

In this remediationprocess, an extracting fluid having chemical reagents such as surfactants, acids, bases, salts, and chelating agents are used for transferring metals from soils to an aqueous solution [24]. The solubility of the metals can be enhanced by leaching agentswhich convert the solid metal into a more soluble form [27,32]. The chemical processes of further five types are depicted in Fig. 10.

#### 2.2.1 Chemical stabilization

In this technique, immobilized agents are used to reducethe movement of HMIsin the soil. The chelating agents desorb heavy metals from the soil by forming strong and water-soluble metal coordination compounds [100]. Further, these chemical agents also assist the adsorption as well as precipitation to immobilizethe contaminants. The chelating agents have many functional groups which are able to donate the nitrogen, sulfur, oxygen and phosphorus atoms for binding with the metal ions.

Manning et al., (2002a) studied that the birnessite (MnO_2_) oxidized the arsenite and reduced the concentration of arsenite in contaminated soil [101]. Kumpiene et al., (2008) observed that iron oxide reduced the concentration of metal (Cu, Pb, Cr, and Zn) and metalloid (As) in polluted soil [102]. Jiang et al., (2011) studied the comparison of chelating agents for the extraction of nickel and copper from contaminated soil [103]. Ullah et al., (2020) used sulfur-containingchelating agentssuch as cysteine, manganesecompounds, zeolite, and iron oxide forthe removal of Cd, As, Cr, and Pb metal ions from paddy soil to enhance rice safety [104].

#### 2.2.2 Treatment with nanoparticles

**Fig. 11:**
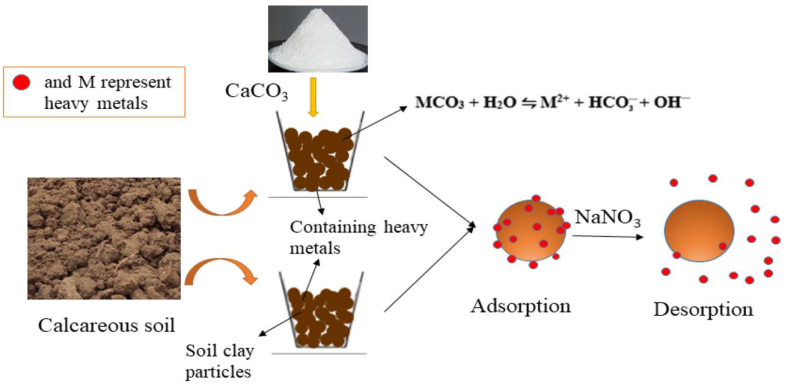
Adsorption of heavy metals from the contaminated soil [Reprinted with permission from the reference number [117] He G, Zhang Z, Wu X, Cui M, Zhang J, Huang X. Adsorption of Heavy Metals on Soil Collected from Lixisol of Typical Karst Areas in the Presence of CaCO_3_ and Soil Clay and Their Competition Behavior. *Sustainability*. 2020; 12(18):7315 Copyright @ MDPI].

Inthe past few years, the use of the nanoparticles technique has been broadlyapplied tothe remediation of metal-contaminated soil. The synthesis of nanoparticles with a diameter of less than 100 nm is feasiblefor the extraction of metal from metal-contaminated soil [105]. The nanoparticles follow various mechanisms such as adsorption, precipitation, redox reactions, and co-precipitation as shown in Fig. 11 [[106], [107], [108], [109], [110]].

Wang et al., (2016) prepared silicon-based nanoparticles for the uptake of cadmium, lead, copper and zinc from metal-contaminated soil [111]. Zhangtao et al., (2017) prepared zeolite-based nanoparticles and removed the Cd(II), Pb(II), and As(III) metal ions from an aqueous solution and soil [112]. Further, Tibor and Melinda (2018) synthesized zero-valent iron nanoparticles for the extraction of heavy metals from metal-polluted sites [113]. Wang et al., (2019) preparedmaize straw-basediron nanoparticles for the remediation of chromium metal from an aqueous solution and soil [114]. Zhang et al., (2020) synthesized modified nanoparticles for the effective trapping of hexavalent chromium metal from polluted soil samples [115]. In addition to this, Zhangtao et al., (2020) prepared zeolite-supported nanoparticles forthe immobilization of Cd, Pb, and As in farmland soils [116].

#### 2.2.3 Stabilization/solidification

**Fig. 12:**
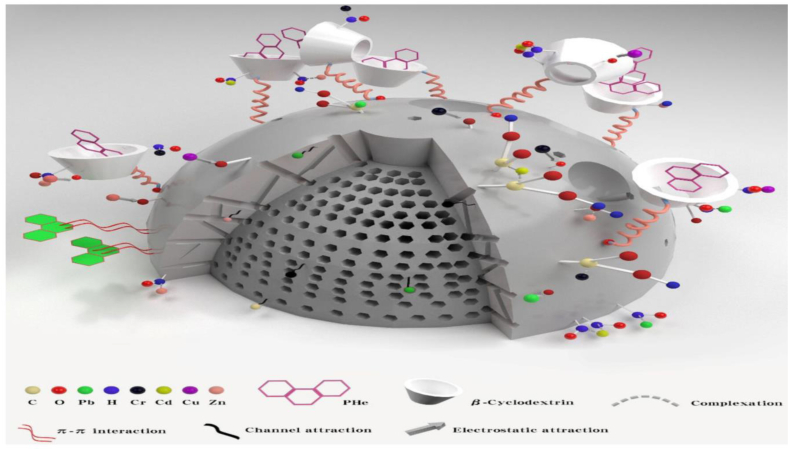
Stabilization/Solidification of Heavy Metals and PHe Contaminated Soil with β-Cyclodextrin Modified Biochar (β-CD-BC) and Portland cement [Reprinted with permission from the reference number [122] Li, G.; Li, H.; Li, Y.; Chen, X.; Li, X.; Wang, L.; Zhang, W.; Zhou, Y. Stabilization/Solidification of Heavy Metals and PHe Contaminated Soil with β-Cyclodextrin Modified Biochar (β-CD-BC) and Portland Cement. *Int. J. Environ. Res. Public Health***2022**, *19*, 1060 Copyright @ MDPI].

In this chemical remediation method, the binding agents viz. asphalt, cement, clay, and fly ash are mixed with the metal contaminated soil to form a stable solid form thatprevents the metal from leaching as shown in Fig. 12. In the first step, the stabilization reduces the movement of the contaminants using a chelating agent, and further, the solidifying agents prevent the contaminantsdiffusion into the environment in the future [35]. This process is less hazardous to the biotic and abiotic systems because chemicals remain in the treated area.

Napia et al., (2012) used zeolite and portland cement as bindersfor the effective immobilization of HMIs from solid waste [118]. Li et al., (2014) observed the use of ordinary portlandcement as a binder in ratios 109/0.2, 83/0.3, and 71/0.4 for leaching out the lead metal fromlead-contaminated soil [119]. Liu et al., (2018) added a thiourea-formaldehyde resin to the polluted soilforadsorbing and stabilizedthe chromium and cadmium. It was observed that this resin was simple, perfect for stabilization, and was non-toxic to the indigenous microorganism as compared to the other agents [120]. Xiaojun et al., (2021) used cementing materials A, as a curing agent for the stabilization and solidification of cadmium, lead, and copper from the metal-pollutedsoil [121].

#### 2.2.4 Chemical soil washing

**Fig. 13:**
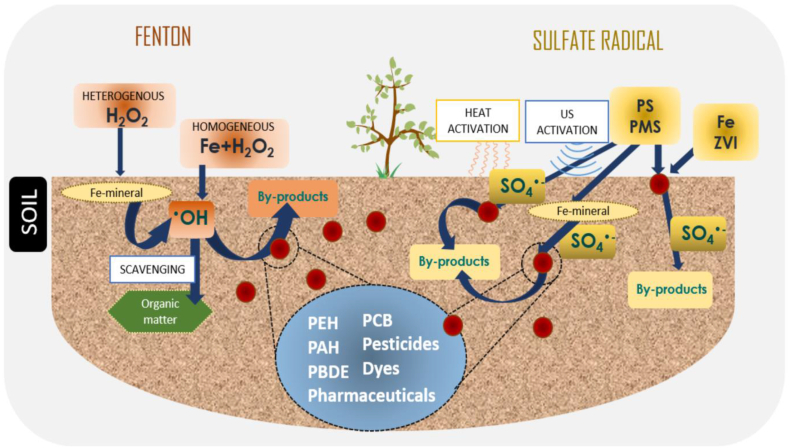
Schematic representation of chemical soil washing using reagents [Reprinted with permission from the reference number [127] Calenciuc, C.; Fdez-Sanromán, A Recent Developments in Advanced Oxidation Processes for Organics-Polluted Soil Reclamation.; Lama, G.; Annamalai, S.; Sanromán, A.; Pazos, M. *Catalysts***2022**, *12*, 64. Copyright @ MDPI].

The soil washing process is an ex-situ remediation process that removes the metal contaminants from the soil by washing with extractant solution viz. water, surfactants, chelators, organic and inorganic acids respectively [37]. The reactants react with these solutions and produce sulfides, carbonates, phosphates, and metal hydroxides as shown in Fig. 13. At the end of the process solid particles can be separated via filtration and sedimentation process. Further, the clean soil may be reused as a backfill at the site.

Zhang et al., (2010) used this method with a chelator for the immobilization of heavy metal from contaminated soil. But this process does not provide satisfying results, because destabilizednature of some strongly bound fractions [123]. Zhai et al., (2018) studied the combined effect ofsoil washing and in situ immobilization (lime, biochar, and carbon black) and reduced 36.5%, 73.6%, 70.9%, and 53.4% bioavailability of Cd, Cu, Pb, and Zn metalsin soil [124]. Feng et al., (2020) applied polyacrylic acid and ethylenediamine tetra(methylene phosphonic acid) for the removal of HMIs from soil in thechemical washing process which decreases the environmental risks and toxicity as compared to other agents [125]. Moreover, Wang et al., (2020) used less toxic and biodegradable acids such as glucomonocarbonic acid, iminodisuccinic acid, polyaspartic acid, and glutamate-*N*,*N*-diacetic acid for extraction of metal from polluted soil. But the use of acids is less effective as compared to ethylene diamine tetraacetic acid (EDTA) washing [126].

#### 2.2.5 Electro-kinetic remediation

**Fig. 14:**
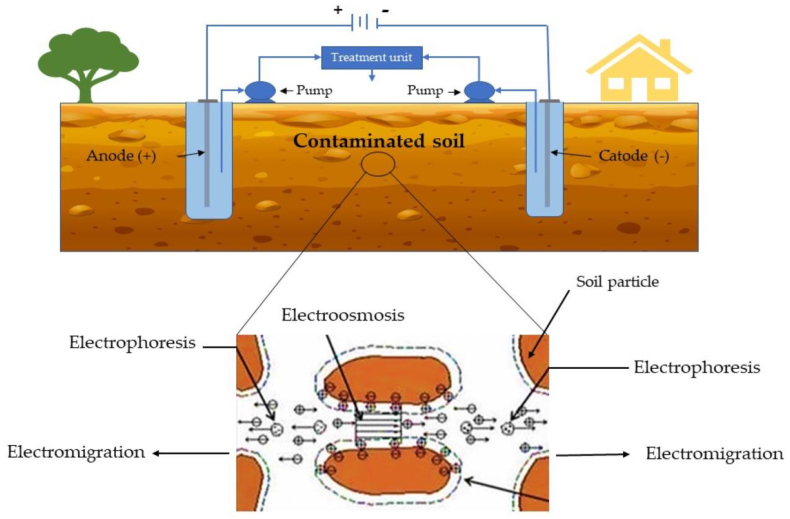
Electro-kinetic remediation process for removal of metal from polluted soil [Reprinted with permission from the reference number [130] Raffa CM, Chiampo F, Shanthakumar S. Remediation of metal/metalloid-polluted soils: A short review. Applied Sciences. 2021 Apr 30; 11(9):4134, Copyright @ MDPI].

In this remediation technique, the direct electric field is applied to migrate the contaminants different metal ionstowardthe oppositely charged electrode. First, the electrodes are dipped in an electrolytic solution and then they are thrusted into the metal-contaminated soil as shown in Fig. 14. The electric field is generated, which migrates the HMIstoward the oppositely charged electrodes [128]. The HMIs pollutantsthat assemble at the electrodes are treated with diverse techniques such asprecipitation, electroplating, and sorption with ion-exchange resins. The various factors affect the metal removal efficiency such asthe nature of the metal contaminants, applied electric field, electrolyte conductivity, and electrode substance etc [129]. The electro-kinetic technique followselectro-migration, electrophoresis, and electro-osmosis mechanisms.

The electro-migration techniqueinvolves the migration of charged species by applying a high-density current across the fluid. The overall electro-migration process depends upon the electric field voltage, electrolyte concentration as well as electric force voltage. In electrophoresis, metal contaminants migrate with charged colloids toward the opposite electrode byapplying the electric field. Further, in the electroosmosis mechanism, a direct electric potential gradient was applied for the movement of metal pollutants with respect to a solid wall. The process mainly depends upon flow rate, because the increase in flow rate enhanced the migration of contaminants as well as removal efficiency [128,129]. Reddyand Cameselle (2009) applied an electro-kinetic technique for the extraction of metals from the metal contaminated soil, sediment, and groundwater [128]. Han et al., (2010) used carbonized food waste as an additive for the removal of copper metal from polluted soil and removed 53.4–84.6% of copper contaminants [42]. Cameselle and Pena et al., (2016) investigated that more than 70% of heavy metals are removed from the soil using citric acids for enhancing the electro-osmosis and electro-migration remediation [39]. Sun et al., (2019) removed 87.60% of Cd metal ions contaminants from soil by applying a superimposed electric field [34]. Yang et al., (2020) studied the combined effect of low molecular weight organic acids and electrochemical for seven days at pH 8.3 and voltage 0.9 V. It was observed that more than 50% of Cu and Zn metal was removed from contaminated soil [40].

#### 2.2.6 Advantages and disadvantages of chemical processes

The chemical remediation processes are a fast, effective and widely used process. Further, the constant flow of the electric field in electrokinetic remediationmakes this process efficient for the extraction of metal from low-permeability soils. However, the presence of chemical agents changes the soil properties as well as produces secondary pollution which impacts biodiversity and different ecosystems.

### 2.3 Biological processes

**Fig. 15:**
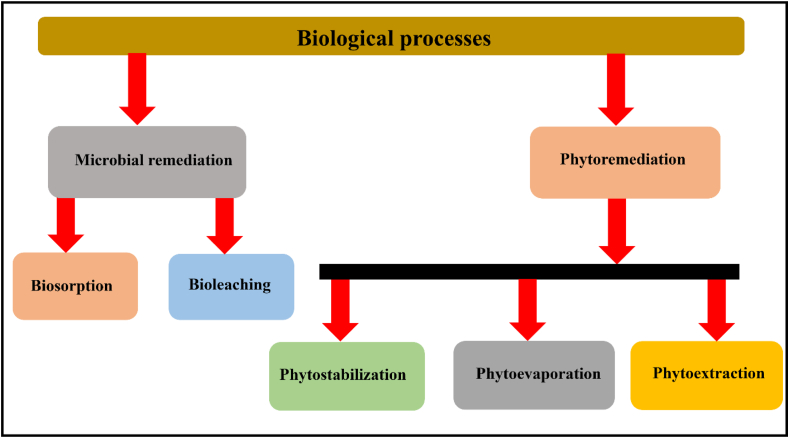
Classification of biological remediation processes for extraction of HMIs from polluted soil.

The biological process is a natural clean-up phenomenon, in which microorganisms and plants are used to remove the metal/metalloids from polluted soil samples [48]. In this technique, metal and metalloids are not directly degraded using plants and microorganisms, butconverted the contaminants into less toxic compounds via various biological processesviz. Enzyme secretion and cellular morphological changes [49]. The biological processescan be further classified into two different processes as shown in Fig. 15.

#### 2.3.1 Microbial remediation

**Fig. 16:**
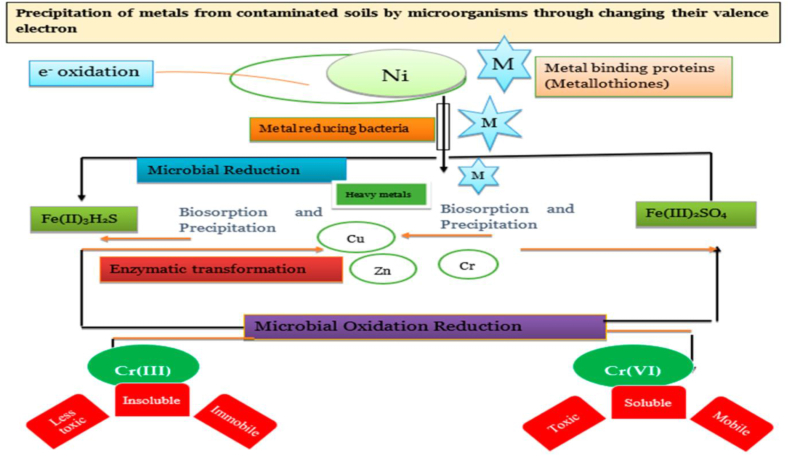
Removal of heavy metals from contaminated soil by microorganisms [Reprinted with permission from the reference number [133] Tarfeen, N.; Nisa, K·U.; Hamid, B.; Bashir, Z.; Yatoo, A.M.; Dar, M.A.; Mohiddin, F.A.; Amin, Z.; Ahmad, R.A.; Sayyed, R.Z. Microbial Remediation: A Promising Tool for Reclamation of Contaminated Sites with Special Emphasis on Heavy Metal and Pesticide Pollution: A Review. *Processes***2022**, *10*, 1358 Copyright @ MDPI].

In this remediation technique, variousmicroorganismssuch as bacteria and fungi are used under aerobic conditions to convert the metal contaminants into less toxic molecules. The microorganisms immobilized, oxidized, reduced, and metabolized the metal and metalloid contaminants as shown in Fig. 16 [131]. Further, the addition of nutrients, fertilizers, and biosurfactants along with microorganisms, increases the metal extraction efficiency. Themicrobial remediations are biosorption and bioleaching [132]. Further, various factors also affect the metal removal efficiency such as pH, temperature, salinity, moisture, types of bacterial species, types of nutrients, and the concentration of contaminants.

##### 2.3.1.1 Biosorption

In this remediation process, various types of biomasses viz. Fungi, bacteria, and algae are used to extract the HMIs from contaminated sites. The metal ionsare immobilized with the cellular structures of the microorganismvia extracellular binding between the metal ions and cell surface [[45], [46], [47], [48], [49], [50], [51], [52],^132^]. The extracellular materials assist the binding mechanism via physical adsorption, complex formation, ion exchange, reduction, and precipitationmethods. Further, various factors affect the metal removal efficiency such as process condition, density of sorption centers, type of metal ion and immobilization agents.

Raghad et al., (2016) studied the bioaccumulation of cadmium and lead metal ions using Shewanellaoneidensis bacteria which was extracted from the Basragovernorate [134]. Zhaojie et al., (2017) investigated the composite microbial agents (Mucor circinelloides, Actinomucorsp, and Mortierella sp) immobilized 74.98%, 85.29%, and 79.41% of zinc, lead, and manganese metals ions from the soil contaminated sites [135]. Bano et al., (2018) used obligate halophilic fungi for the biosorption of heavy metals ions and found that 86% and 83% of heavy metals adsorbed inthe case of Aspergillus flavus and Sterigmatomyceshalophilus fungi [48]. Hassan et al., (2019) observed 62%, 59%, 49%, 42%, and 38% of As, Mn, Cu, Cr, and Fe metal removal efficiency from polluted soil using filamentous fungi consortia [49].

##### 2.3.1.2 Bioleaching

In this process, the mobility and stability of the heavy metal ions are reducedby microorganismsas they produce secretions such as organic acids which easily dissolve the metal, metalloids, and soil particles [53]. The metal ionsare made directly soluble via the metabolism of microorganisms. The various agents such as lipids, polysaccharides, lipopeptides, and biosurfactants show high surface activity that increases the binding affinity of metal ions and the chelate formation capacity [54].

Yang et al., (2017) investigatedthe effect of oilseed sunflower, peanut, and sesame for the extraction of cadmium and lead ions from the soil. It was observed that a maximum of 458.6 g/ha and 1264.7 g/ha of cadmium, and lead respectively were obtained using sunflower [53]. Peng et al., (2019) used Aspergillus flavus fungi for the bioleaching of metals in contaminated soil and found that bioleaching efficiencies were 16.91%, 49.66%, and 65.73% for lead, cadmium, and zinc respectively [136]. Further, Gorecka et al., (2021) investigated the effect of various variants of bioleaching with bacteria and obtained bioleaching efficiency of 66% of zinc after 14 days and 99% of cadmium after 7 days of the leading process [137]. Sur et al., (2022) used the Thiobacillus ferrooxidans bacteria for the bioleaching of copper, lead, chromium, and nickel. It was observed that maximum bioleaching efficienciesof 76%, 32%, 72%, and 68% of copper, lead, chromium, and nickelwere achieved after 12 h of the treatment process [138].

#### 2.3.2 Phytoremediation

**Fig. 17:**
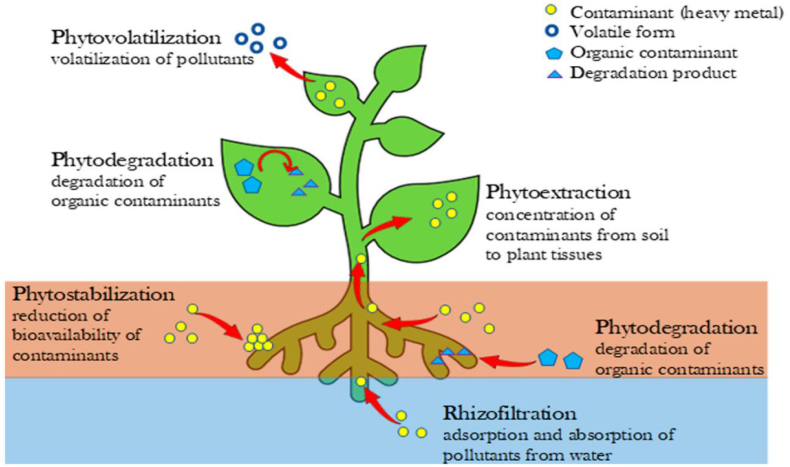
Phytoremediation process for metal-polluted soil. [Reprintedwithpermission from the reference number [141]Rigoletto M, Calza P, Gaggero E, Malandrino M, Fabbri D. Bioremediation Methods for the Recovery of Lead-Contaminated Soils: A Review. *Applied Sciences*. 2020; 10(10):3528 Copyright @ MDPI].

In phytoremediation, specific types of plants are used toadsorb, transfer, and stabilize the metal contaminants from the soil as shown in Fig. 17. The plant roots have certain enzymes which accumulatethe metal and metalloids from the soil and deposit them in the plant biomass above the soil [139]. Phytoremediation is a slowprocess, if the plant has large roots and shoots then it can easily accumulate high quantities of metal ions from the soil [140]. Nwaichi et al., (2016) studied that some plants are called hyperaccumulators, which collectgreater than 1000 mg/kg ofCo, Pb, Cr,Ni, Cd, and Co ions from the polluted soils [139]. Inthe last few years, many plants and agricultural/herbaceous crops are applied viz. willow, poplar, wheat, smilo grass, sweet, and grain sorghum respectively for the removal of metal/metalloids from the soil. Further, phytoremediation depends upon the types of plants and contaminants it is of three typesphytostabilization, photoevaporation, and phytoextraction.

##### 2.3.2.1 Phytostabilization

In this process, plants bioaccumulate HMIs by reducing the movements of HMIs in the soil. The plant's roots have certain binding molecules viz. phytochelatins and metallothioneins which make a complex with the metal ions and extract the metal from the metal ions contaminated soil [142]. Dary et al., (2010) studied the metal phytostabilization using Lupinus luteus inoculated with Bradyrhizobium sp. 750 and observed that bacterial species increased both nitrogen content and biomass which enhanced the metal ions removal efficiency [143]. Galal et al., (2017) used the vossiacupidata as a phytoremediator forthe remediation of metal from the metal contaminated water bodies [144]. Saran et al., (2020) investigated the effect of the Helianthus petiolaris plant on the uptake of cadmium and lead metals from metal-pollutedsoil and observedthat it is efficient for less than 50 mg/kg and 1000 mg/kg of cadmium and lead concentration respectively in soil [145]. Li et al., (2021) investigated the effect of manures and compost fertilizers on halophytic plants for the biosorption of heavy metals ions from agricultural soil and found that fertilizer enhanced the metal extraction efficiency of zinc, copper, cadmium, and lead respectively [146].

##### 2.3.2.2 Phytoevaporation

Phytoevaporation involves the uptake of heavy metal and metalloid contaminants by plant roots and evaporates into the atmosphere through the transpiration process. This process is widely used for the extraction of highly volatile metals such as selenium and mercury from metal-polluted sites [70]. From the past few years studies, it was found that Astragalus racemosus and Arabidopsis thaliana plants enhance the volatility of metals, and convert the selenium into dimethyl diselenide and mercury (II) to mercury [69]. Leonard et al., (1998) used five plant species viz. Lepidium latifolium, Artemisia douglasiana, Fragaria vesca, Caulanthus species, and Eucalyptus globulus for comparison and observed that the Caulanthus species shows 92.6 mg/m^2^. h higher mercury emission rate as compared to other species [147]. Sakakibara et al., (2010) investigated the use of the Pteris vittate plant to convert arsenic into volatile arsenite and removed 90% of arsenic concentration from contaminated soil [70]. Wang et al., (2012) studied mercury metal extraction from contaminated soil using different types of plant species [148]. Li et al., (2022) observed that the addition of 15 g/kg of modified vermiculite-montmorillonite reduces 98.2% mercury concentration from polluted soil samples [149].

##### 2.3.2.3 Phytoextraction

Phytoextraction is a subprocess of phytoremediation in which HMIs are absorbed by the plant roots and deposited in the aboveground, harvestable biomass [[66], [67], [68]]. This technique consists repeated harvesting of the biomass in order to lower the concentration of the metal pollutants from the soil. Mc-Grath and Zhao (2003) studied thehyperaccumulation of zinc, cadmium, nickel, and arsenic from polluted soil sites using plants and chelating agents [150]. Iqbal et al., (2015) investigated the phytoextraction capacity of a plant speciesand found that phytoextraction increases chelating agents along with protoplast fusion, hybridization and genetic engineering [151].

Agnello et al., (2016) studied the combined effect of phytoextraction and bioaugmentation using Medicago sativa L plant and Pseudomonas aeruginosa bacterial strain for remediation of zinc, copper, and lead from contaminated soil [152]. Mahmood -ul-Hassan et al., (2020) did a study of various non-eatable floriculture plants viz. calendula, antirrhinum, marigold, and pansy for extraction of Cd, Cr, Ni, and Pb from polluted soil. It was observed that the addition of bacterial inoculum and EDTA with these plants enhanced the transformation of the metal ions from the root to the shoot systems [153].

Hence it is concluded that phytoremediation is often combined with other remediation techniques such as bioaugmentation, EDTA-amended soil, electrokinetic etc, which enhanced the metal ions removal efficiency in phytoremediation.

#### 2.3.3 Advantages and disadvantages of biological process

The biological process is a quite successful technique, remediating the metal contaminants without leaving any harmful by-products. Further, this process is cost-effective in nature and removes metal/metalloid pollutants without disturbing the surrounding environment. However, this process is slow and time-consuming that needs several months to years for a satisfactory removal efficiency result. Further, the various factors such as pH, salinity, temperature, and nature of the soil hinder the microorganism growthfor remediation ofHMIs/metalloids.

## 3.0 Combined remediation process

The different individual remediation technologiesemployed for the removal of HMIs from the contaminated soilface some critical challenges like expensiveness, time-consuming, effect on soil characteristics, produces secondary pollution as well as in-situ treatment failure. Hence, there is a need of combined or integrated remediation technology which can overcome these drawbacksand be applied ona large scale. Basically, an integrated remediation process is the combination of two or more remediation processes that not only increased the removal efficiency and rate but also removed the additional contaminants along with HMIs from the contaminated soils. In this review article we have discussed three combination technologies viz. the chemical-biological remediation process, electrokinetic-microbial remediation process and electrokinetic-phytoremediation process.

### 3.1 Chemical-biological remediation process

This integrated process involves chemical treatment followed by biological treatment and vice-versa, which acts as aenhancing step due to its economic feasibility as well as effectiveness. As reported by many researcher groups, implementation of this integrated treatment process provides a significant result for HMIs as compared to indivisible biological and chemical treatment processes [[154], [155], [156], [157], [158]]. Sharma and Malaviya (2014) studied the combined effect of biological system using Fusarium chlamydosporium and chemical precipitation to reduce the chromium metal ion concentration (62.33%), turbidity (64.69%) and chemical oxygen diamond (71.80%) of metal contaminated soils [159]. Similarly, Ahmed et al., (2016) used the biological treatment and chemical precipitation for the removal of chromium metal from tannery effluent. It was observed that 98.4% and 99.3% of Cr(VI) and Cr (III) metal ions were recovered respectively. Along with this, it also reduces 77% and 81% of chemical oxygen demand and turbidity [160]. This method is considered a highly cost-effective as well as eco-friendly alternative to the single remediation technologies for uptakeof HMIs from contaminated soil sites.

### 3.2 Electrokinetic-microbial remediation process

In this integrated technique, the soil contaminants are electrochemically converted into useful by-products and produce bioelectricity via microbial metabolic processes [161]. This process was reported to promote the bioavailability of the pollutants, increased the biodegradation efficiency through oxidation and reduction zones, improved nutrient transport, extraction of soil pollutants, and availability of electron acceptors [162].

The biological system produces both alkalophilic and acidophilic microbes. The acidic bacteria help in electrokinetic, whereas alkalophilic favor in metal precipitation. Peng et al., (2011) used indigenous iron-oxidizing bacteria and electrokinetics for the reduction of zinc and copper metal from 375.6 mg/kg to 33.3 mg/kg and 296.4 mg/kg to 63.4 mg/kg respectively [163]. Rosestolato et al., (2015) removed 60% mercury metal ions from 400 kg of contaminated soil using a bio-electrokinetic integrated technique [164]. Further, Azhar et al., (2016a) studied that 78% of mercury metal ion is removed using 50 V electric current and Lysinibacillus fusiformis bacteria for seven days in a bio-electrokinetic combined method [165]. In addition to this, Azhar et al., (2016b) also did another study for the removal of zinc metal from contaminated soil using Pseudomonas putida with electrokinetics and showed 89% of metal removal within 5 days [166]. Due to the unique characteristic viz. high removal efficiency, energy saving and no secondary pollution, nowdays the electrokinetic-bioremediation combined process has been widely employed for remediation of soil contaminants.

### 3.3 Electrokinetic-phytoremediation process

This combined remediation method involves electrokinetic treatment followed by phytoremediation treatment and vice-versa. In electrokinetics, a direct electric current passed between the electrodes which separated the metal contaminants from soil [167]. But,thephytoremediation depends upon the plants uptake mechanism viz. phytostabilization, phytoextraction, photoevaporation, rhizofiltration, and rhizodegradation [[168], [169], [170]]. In combined electrokinetic-phytoremediation the low intensity electric field is applied in the vicinity of the growing plants in the polluted soil, which increase the bio-avaibility of the metal contaminants are high. Further, this integrated approach provides effective results in terms of metal recoveryand is more economical as compared to the other integrated methods. Lim et al., (2004) used the electrokinetic-phytoremediation integrated method for the removal of lead metal from polluted soil and obtained 2–4 times more effective removal as compared to the indivisible used these methods [171]. In 2012, Cang et al., employed an electrokinetic-assistedphytoremediation technique and extracted the Zn, Cu, Cd, and Pb metal ions from the contaminated soil. It was noticed that the properties of soil are directly affected via the voltage applied but the plant growth increased the enzymatic activities which increased the heavy metal removal efficiency [172]. In addition to this, Kubiak et al., (2012) have remediated 90% toxic arsenic metal from the artificial water sample which was prepared using 150 μg/L concentration of sodium arsenate [173].

Similarly, Bhargavi and Sudha (2015) removed 59.78% and 67.43% of Cr (VI) and Cd (II)metal ionsfrom Ranipet industrial area using 50 V electric current and Brassica Juncea plant after 25 days of treatment in electrokinetic-phytoremediation technique [174]. Further, Mao et al., (2016) applied an electrokinetic enhanced phytoremediation technique for the extraction of As, Pb and Cs heavy metals from polluted soil by lowering the pH of soil up to 1.5 and increasing the solubility and bioavailability of the heavy metals [155].

## 4.0 Case studies

The twelvediverse case studiesare shown in Table 3. In these case studies, different locations were opted, from eight different countries and various techniques were applied to clean the metal-contaminated soils. The area of treatment was varied from a few centimetres to hundred meters to check the viability of the processes. It was observed that around the whole world, efforts were made in one or the other way to clean the metal-contaminated sites. Further, it was also noticed the selection of the technologies wasdifferent factor-dependent. In comparison to the single technology used the combination of two or more technologies showed better results. In nutshell, it could be summarised that the combination of technologies are better option for cleaning HMIs contaminated soils.

**Table 3:** Different locations case studies for HMIs.

Remediation technique	Type of Contaminants	Year	Size	Location	Reference
Electrokinetic remediation	Pb, Cu, Ni, Zn	1997	57 m^2^	Paducah, Kentucky	[175]
Chemical stabilization	Zn, Cu, Cr, Ni, Co, Hg, Cd, As, Pb	2006	0–25 cm	Katterdan, India	[176]
Phytoremediation	As, Zn, Cd, Pb	2008	1000 m^2^	Porto Marghera, Italy	[177]
Soil washing and phytoremediation	Cu, Zn, Cd, Pb	2011	64 m^2^	China	[178]
Chemical stabilization	Cd, Zn, Pb	2012	10 m^2^	Biscay, Spain	[179]
Phytoremediation	Zn, Cr, Cd, Pb, As	2013	1600 m^2^	Taranto, Italy	[55]
Electrokinetic remediation	Pb, Cu, As	2013	26.25 m^3^	Janghang, South Korea	[180]
Phytoremediation	Hg, Pb, Zn, Cu, Cd, Fe, Ag, As	2013	Dna	Agra, India	[181]
Soil replacement	Cd, Hg, Cr, Cu, Ni, Pb, Zn, As, Ba, Be, Sb, Se, Mo	2014	Dna	Serbia	[182]
Electrokinetic-phytoremediation	Cr, Cd	2015	15–30 cm	Ranipet, Tamilnadu, India	[174]
Electrokinetic remediation	Cr, Co, Cu, Ni, Pb, Zn	2020	0–15 cm	Gurugram, India	[183]
Electrokinetic-bioremediation	Cu, Pb, Ni	2021	Dna	Xuzhou, China	[184]

Dna: Data not available.

## 5.0 Conclusion and future perspectives

The HMIs contamination in soil is a quite serious matter of concern. For fast and effective metal extraction, first, we need to understand the type and concentration of the metal in the soil which helps in better selection of remediation technology. Further, various characteristics of the soil such as type, permeability, and pH also affect the metal clean-up capacity from contaminated soil. Hence, it was observed that the choice of remediation process depends upon the metal and soil types, as the incorrect selection may prevent high removal efficiency. Various types of remediation processes such as physical, chemical, biological, and combined remediation techniques have been proposed, studied, and applied for the removal of heavy metals in the last few years but there are still a few limitations and challenges need to be addressed in future.

The physical methods, are the easiest methods for the removal of HMIs from polluted soil but are labor-intensive, time-consuming, and expensive in comparison to chemical methods and biological methods. The chemical methods are quite fast, with high metal uptake efficiency but are expensive, non-eco-friendly, and are not practically applicable for large-scale applications.

The biological methods are quite eco-friendly butthey require specific types of microorganisms such as bacteria and fungi and their growth conditions for the removal ofa particular type of heavy metal contaminants. In phytoremediation methodsare simple and cost-effective as compared to the others but it takes years to grow a particular type of plant for remediation of contaminated soil.

Keeping the future perspectives in mind, we can suggest thatthe solution to all the above limitations by employing different technologies in combinations as a single process. To elaborate it, the combination can be made by opting the best part of the technology and more than one technology can be employed simultaneously achieving the desired results.

## Author contribution statement

All authors listed have significantly contributed to the development and the writing of this article.

## Data availability statement

Data will be made available on request.
